# Genetic and epigenetic control of plant heat responses

**DOI:** 10.3389/fpls.2015.00267

**Published:** 2015-04-24

**Authors:** Junzhong Liu, Lili Feng, Jianming Li, Zuhua He

**Affiliations:** ^1^National Laboratory of Plant Molecular Genetics, Institute of Plant Physiology and Ecology, Shanghai Institutes for Biological Sciences – Chinese Academy of SciencesShanghai, China; ^2^School of Life Science and Technology, ShanghaiTech UniversityShanghai, China; ^3^Plant Signaling Laboratory, The Plant Stress Biology Center, Shanghai Institutes for Biological Sciences – Chinese Academy of SciencesShanghai, China

**Keywords:** heat, genetic mechanism, epigenetic regulation, small RNAs, transgenerational memory

## Abstract

Plants have evolved sophisticated genetic and epigenetic regulatory systems to respond quickly to unfavorable environmental conditions such as heat, cold, drought, and pathogen infections. In particular, heat greatly affects plant growth and development, immunity and circadian rhythm, and poses a serious threat to the global food supply. According to temperatures exposing, heat can be usually classified as warm ambient temperature (about 22–27°C), high temperature (27–30°C) and extremely high temperature (37–42°C, also known as heat stress) for the model plant *Arabidopsis thaliana*. The genetic mechanisms of plant responses to heat have been well studied, mainly focusing on elevated ambient temperature-mediated morphological acclimation and acceleration of flowering, modulation of circadian clock and plant immunity by high temperatures, and thermotolerance to heat stress. Recently, great progress has been achieved on epigenetic regulation of heat responses, including DNA methylation, histone modifications, histone variants, ATP-dependent chromatin remodeling, histone chaperones, small RNAs, long non-coding RNAs and other undefined epigenetic mechanisms. These epigenetic modifications regulate the expression of heat-responsive genes and function to prevent heat-related damages. This review focuses on recent progresses regarding the genetic and epigenetic control of heat responses in plants, and pays more attention to the role of the major epigenetic mechanisms in plant heat responses. Further research perspectives are also discussed.

## Introduction

Owing to the global warming, the annual mean maximum and minimum temperatures have been reported to increase by 0.35 and 1.13°C, respectively, for the period 1979–2003 (Peng et al., 2004). It is probable that the growing season temperatures in the tropics and subtropics by the end of the 21st century will exceed the most extreme seasonal temperatures recorded from 1900 to 2006 (Battisti and Naylor, 2009). Global warming has profound and diverse effects on plants. Warmer temperature has advanced the average first flowering date of 385 British plant species by 4.5 days (Fitter and Fitter, 2002). Besides, recent climate warming (2001–2008) has shifted vascular plant species’ ranges to higher altitudes in European mountainous regions (Pauli et al., 2012). Furthermore, it is noteworthy that climate warming poses a serious threat to the global crop yields. Over the past three decades (1980–2008), heat has caused a decrease of 3.8 and 5.5% in the global maize and wheat production (Lobell et al., 2011). It is estimated that global yields of the six most widely grown crops (wheat, rice, maize, soybeans, barley, and sorghum) drop by 0.6 ∼ 8.9% for every 1°C the temperature increases (Lobell and Field, 2007). In the dry season, global warming has been estimated to cause a ∼10% reduction in rice yield for every 1°C increase in growing-season minimum temperature (Peng et al., 2004). Thus, it is critical to dissect the heat sensing and signal transduction pathways in plants.

Genetic knowledge of plant responses to heat stress has been accumulating, including several putative heat sensors, HSFs and HSPs (heat shock factors and proteins) response pathways, and the network of phytohormones, chaperones, and secondary metabolites (Bokszczanin and Fragkostefanakis, 2013; Qu et al., 2013). However, our understanding of plant responses to warm temperature is limited, despite recent discoveries implicating the central role of the basic helix-loop-helix (bHLH) transcription factor *PHYTOCHROME INTERACTING FACTOR 4* (*PIF4*) in warmth-mediated morphological acclimation and acceleration of flowering (Proveniers and van Zanten, 2013). The modulation of circadian clock and immunity by high temperature also remains largely unknown. Recently, epigenetic regulations of heat responses have attracted increasing interests. The epigenetic mechanisms in response to heat include covalent modifications of DNA and histones, histone variants, ATP-dependent chromatin remodeling, histone chaperones, small RNAs, long non-coding RNAs (lncRNAs), and other undefined mechanisms (**Table 1**). This review briefly introduces the genetic mechanisms of plant responses to heat and highlights recent progresses regarding the underlying epigenetic regulations mainly in the *Arabidopsis* model, with aspects of some important physiological processes.

**Table 1:** Different epigenetic regulations involved in different heat responses.

Plants	Heat treatment	Major effects	Major epigenetic regulations	Reference
*Saccharina japonica*	20°C for 3 h	Regulation of tolerance to heat stress	miRNAs	Liu et al. (2014)
*Arabidopsis thaliana*	Grown at 26°C	Elevated survival of *Turnip Crinkle Virus*-infected Plants	siRNAs	Zhang et al. (2012)
*A. thaliana*	Grown at 27°C	Early flowering, hypocotyl and petiole elongation	H2A.Z	Kumar and Wigge (2010), Kumar et al. (2012)
*Nicotiana benthamiana*	Grown at 27°C	Enhanced antiviral defense	siRNAs	Qu et al. (2005)
*N. benthamiana Manihot esculenta*	Grown at 25–30°C	Enhanced antiviral defense	siRNAs	Chellappan et al. (2005), Andika et al. (2013)
*A. thaliana*	Grown at 30°C	Warmth-induced PTGS release with transgenerational memory	miRNAs and siRNAs	Zhong et al. (2013)
*Oryza sativa*	34°C for 48 h	Smaller seed size	DNA methylation, H3K9me2	Folsom et al. (2014)
*Hordeum vulgare*	35.5°C for 24 h	Regulation of tolerance to heat stress	miRNAs	Kruszka et al. (2014)
*Gossypium hirsutum*	35–39°C /29–31°C day/night for 7 days	Regulation of anther development	DNA methylation, histone modifications	Min et al. (2014)
*A. thaliana*	37°C for 3 h/day during the day for 1 week	Increase in homologous recombination frequency with transgenerational memory	DNA methylation, small RNAs	Boyko et al. (2010)
*A. thaliana*	37°C for 1–4 h	Regulation of tolerance to heat stress	Ta-siRNAs and miRNAs	Guan et al. (2013), Li et al. (2014b)
*Populus tomentosa*	37°C for 8 h	Regulation of tolerance to heat stress	miRNAs	Chen et al. (2012)
*A. thaliana*	37°C for 12 h	Regulation of tolerance to heat stress	long non-coding RNAs	(Di et al., 2014)
*A. thaliana*	37°C for 12 h	Heat stress-induced alternative splicing of *miR400*	*MiR400*	Yan et al. (2012)
*A. thaliana*	37°C for 16 h	Mediating the temporary growth arrest	ATP-dependent chromatin remodeling	Mlynarova et al. (2007)
*A. thaliana*	37°C for 24 h	Transgenerational retrotransposition of ONSEN	siRNAs	Ito et al. (2011)
*Helianthus annuus*	37°C for 24 h	Regulation of tolerance to heat stress	*miRNA396*	Giacomelli et al. (2012)
*P. trichocarpa*	37°C for 24 h	Regulation of tolerance to heat stress	miRNAs	Lu et al. (2008)
*M. esculenta*	37°C for 24 h	Regulation of tolerance to heat stress	miRNAs	Ballen-Taborda et al. (2013)
*A. thaliana*	37°C for 30 h	Release of TGS with reduced nucleosome occupancy and loss of chromocenter organization	CAF-1-dependent chromatin assembly complex	Pecinka et al. (2010)
*A. thaliana*	4°C for 1 week and then 37°C for 15 h	Release of TGS	Unorthodox and potentially new mechanisms	Tittel-Elmer et al. (2010)
*A. thaliana*	37°C for 4 days or 44°C for 30 min	Reorganization of chromatin and release of transcriptional gene silencing,	HIT4-dependent TGS regulation pathway	Wang et al. (2014)
*A. thaliana*	38°C for 1 h	Down regulation of *HSFB2a* involved in gametophyte development	Long non-coding antisense RNA *asHSFB2a*	Wunderlich et al. (2014)
*Apium graveolens*	38°C for 1 h	Regulation of tolerance to heat stress	miRNAs	Li et al. (2014a)
*A. thaliana*	38°C for 1.5 h per day in the dark and then returned to normal growth conditions; for 7 consecutive days	Modulation of pattern-triggered immunity	Histone modifications	Singh et al. (2014)
*A. thaliana*	Several heat cycles (37°C for 12 h in the light and 21°C for 12 h in the dark)	Activation of the imprinted gene *SDC*	An undefined epigenetic mechanism	Sanchez and Paszkowski (2014)
*Triticum aestivum*	40°C for 1 h	Regulation of tolerance to heat stress	miRNAs and long non-coding RNAs	Xin et al. (2010), Xin et al. (2011)
*Chlamydomonas reinhardtii*	40°C for 1 h, for three times	Regulation of tolerance to heat stress	Histone modifications and chromatin remodeling	Strenkert et al. (2011)
*O. sativa*	42°C day/36 °C night for 24 h	Regulation of tolerance to heat stress	miRNAs	Sailaja et al. (2014)
*T. aestivum*	42°C for 2 h	Regulation of tolerance to heat stress	miRNAs	Kumar et al. (2014)
*Brassica rapa*	42°C for 3 h per day for 7 days	Stress-induced transgenerational inheritance	*miR168* and *braAGO1*	Bilichak et al. (2015)
*A. thaliana*	42°C for 16 h	Transcriptional reprogramming	DNA methylation, histone acetylation	Popova et al. (2013)
*A. thaliana*	42°C for 48 h	Stress-induced release of GUS silencing	H3K9ac1 and H3K9/14ac2	Lang-Mladek et al. (2010)
*A. thaliana*	BT and AT^a^	Gene transcription activation	Histone chaperone ASF1	Weng et al. (2014)
*A. thaliana*	BT and AT^a^	Regulation of tolerance to recurring environmental stress	The *miR156-SPL* module	Stief et al. (2014)
*Brassica rapa*	46°C for 1 h	Regulation of tolerance to heat stress	miRNAs, nat-siRNAs, chloroplast small RNAs	Wang et al. (2011), Yu et al. 2012b, (2013)
*A. thaliana*	50°C for 3 h/day for 5 day	Transgenerational phenotypic and epigenetic changes	H3K9 methylation and DNA methylation	Migicovsky et al. (2014)
*Quercus suber*	Temperature increases by 10°C every 3 days from 25–55°C	Acclimation to high temperature	DNA methylation, histone acetylation	Correia et al. (2013)

*^a^BT, basal thermotolerance, 45°C for 2 h; AT, acquired thermotolerance, pretreated at 37°C for 1–1.5 h and returned to 22°C for several hours or days for recovery, treated at 45°C for 2 h*.

## Genetic Mechanisms of Plant Responses to Heat

### Warm Temperature-Mediated Morphological Acclimation and Acceleration of Flowering

**FIGURE 1:**
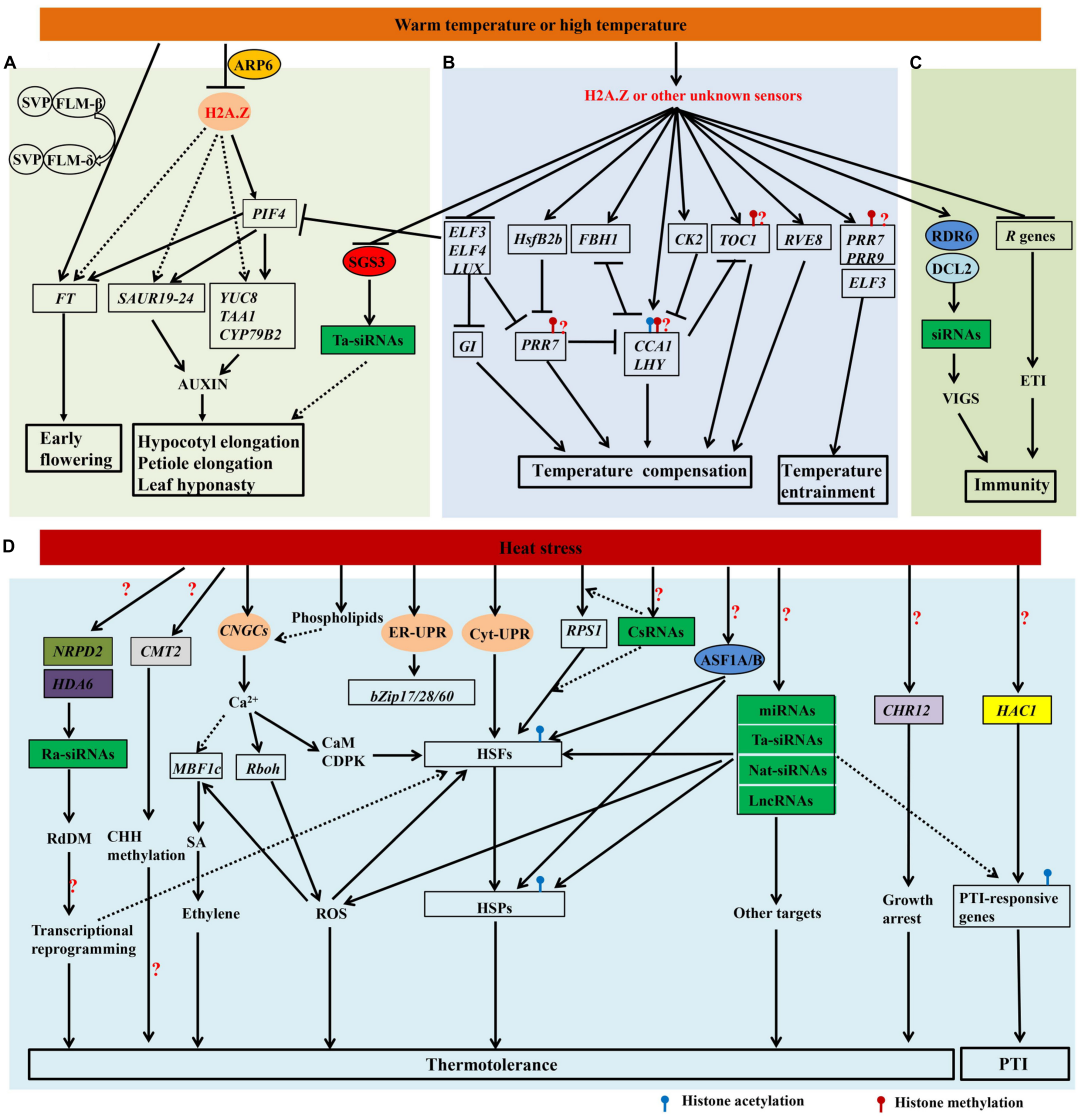
Proposed model integrating genetic and epigenetic controls of heat responses. Genes and proteins are represented in boxes and circles, respectively. The genes and proteins in color are involved in the epigenetic regulation of heat responses. The four putative heat sensors, H2A.Z, the calcium channel in the plasma membrane (*CNGCs*), two unfolded protein sensors in ER (ER-UPR) and the cytosol (Cyt-UPR), are indicated. The speculative regulatory paths are indicated with broken arrows. **(A)** Warm temperature mediates the morphological acclimation and acceleration of flowering. Under warm temperatures, the expression of *PIF4* could be induced by the eviction of H2A.Z at its promoter. PIF4 binds to the promoters of target genes and plays a central role in the morphological acclimation and acceleration of flowering. Warm temperature also induces the transition from SVP-FLM-β to the competing SVP-FLM-δ complex, the latter is then released from the promoter of *FT*. The inhibition of ta-siRNAs (green box) through the down-regulation of SGS3 protein (red circle) by warm temperature may be also involved in the morphological acclimation. **(B)** The genetic mechanisms of temperature entrainment and temperature compensation are proposed. *ELF3*, *PRR7* and *PRR9* are involved in temperature entrainment, while *CCA1*, *LHY*, *PRR7*, *PRR9*, *GI*, *CK2*, *RVE8, FBH1* and *HsfB2b* are proved to play roles in temperature compensation. Note that histone modifications of *LHY*, *CCA1*, *TOC1*, *PRR7* and *PRR9*, such as H3K56ac, H3K9/14ac, H3K4me3 and H3K4me2, may (question mark) be regulated by high temperatures. **(C)** High temperature inhibits *R* genes-mediated ETI and enhances RNA-silencing mediated resistance. Reduced H2A.Z-containing nucleosome occupancy or other unknown mechanism are likely involved in the modulation of clock **(B)** and immunity **(C)**. **(D)** Heat sensors and main signal transduction pathways in heat stress responses (HSR) are shown. Heat stress activates CNGCs, ER-UPR, and Cyt-UPR, and triggers signaling through multiple kinases as well as transcriptional regulators of the HSR, such as HSFs, MBF1c, and Rboh. RPS1 in the chloroplast also responds to heat stress, and generates a retrograde signal to activate *HsfA2*-dependent heat-responsive genes in the nucleus. Some csRNAs are highly sensitive to heat stress and may regulate *RPS1*-mediated heat stress responses. Heat stress also affects the production of some ra-siRNAs, miRNAs, ta-siRNAs, nat-siRNAs, and lncRNAs. These non-coding RNAs may regulate *HSFs*, *HSPs*, and other target genes that function in heat acclimation. The *NRPD2* (olive box) and *HDA6* (purple box)-dependent RdDM pathway and the *CMT2* (gray box)-dependent CHH methylation may be required for thermotolerance. AtASF1A/B proteins (blue circle) are recruited onto chromatin and facilitate H3K56ac, which promotes the activation of some *HSFs* and *HSPs*. The chromatin-remodeling gene *CHR12* (light purple box) plays a vital role in mediating the temporary growth arrest of *Arabidopsis* under heat stress. Repetitive heat stress has also been reported to modulate PTI in a *HAC1* (yellow box)-dependent manner. Many unknown steps (?) remain to be recognized in this model.

The responses of *Arabidopsis* plants to warm temperature include hypocotyl and petiole elongation, leaf hyponasty, and early flowering (Gray et al., 1998; Balasubramanian et al., 2006; Koini et al., 2009). Warm temperature promotes auxin accumulation and activate the gibberellin (GA) and brassinosteroids (BRs) pathway resulting in hypocotyl elongation (Gray et al., 1998; Stavang et al., 2009). PIF4 plays a central positive role in the acclimation to increased ambient temperature (**Figure 1A**; Proveniers and van Zanten, 2013). Warm temperature induces transient expression of PIF4 (Koini et al., 2009; Kumar et al., 2012). PIF4 has been demonstrated to control morphological acclimation to warm temperature via auxin. PIF4 binds to the promoters of the key auxin biosynthesis genes in a temperature-dependent manner (Franklin et al., 2011; Sun et al., 2012). PIF4 may also target the auxin-responsive gene *INDOLE-3-ACETIC ACID INDUCIBLE 29* (*IAA29*) at warm temperature (Koini et al., 2009). Moreover, PIF4 directly or indirectly stimulates the expression of auxin target genes *SMALL AUXIN UP RNA* (*SAUR*) *19–24*, which drive warmth-induced hypocotyl elongation and probably petiole elongation and leaf hyponasty (Franklin et al., 2011). In addition to morphological acclimation, PIF4 controls warm temperature-mediated floral induction through direct activation of the floral pathway integrator gene *FLOWERING LOCUS T* (*FT*) by binding its promoter (Kumar et al., 2012). A receptor-like kinase SCRAMBLED/STRUBBELIG (SCM/SUB) also plays a role in coordinating cell proliferation and differentiation during leaf development under increased ambient temperature (Lin et al., 2012).

Besides PIF4, MADS-box genes *SHORT VEGETATIVE PHASE* (*SVP*) and *FLOWERING LOCUS M* (*FLM*)/*MADS AFFECTING FLOWERING* (*MAF*) *1–5* modulate flowering time in response to ambient temperature changes (Balasubramanian et al., 2006; Lee et al., 2007; Gu et al., 2013). These genes act as flowering repressors and loss of their function leads to accelerated flowering independent of the photoperiod pathway. Interestingly, the RNA processing-related gene products are enriched upon thermal induction, suggesting that temperature might affect RNA processing in *Arabidopsis*. For instance, *FLM* is subject to temperature-dependent alternative splicing (Balasubramanian et al., 2006). The SVP-FLM-β complex is predominately formed at 17°C and prevents precocious flowering. By contrast, the competing SVP-FLM-δ complex is impaired in DNA binding and acts as a dominant-negative activator of flowering at 27°C (**Figure 1A**; Lee et al., 2013; Pose et al., 2013). Therefore, *PIF4* is an activator of *FT* while the MADS-box genes are repressors. However, how the two antagonistic pathways are integrated to modulate flowering time at warm conditions still needs to be genetically dissected.

### The Effect of High Temperature on Circadian Clock

As a cellular time-keeper mechanism, the circadian clock allows plants to coordinate environmental time cues, such as photocycles (light/dark) and thermocycles (warm/cold), with endogenous biological rhythms with a period of ∼24 h. Recent studies have suggested that plant circadian clock consists of three interlocked transcriptional feedback loops, i.e., a core oscillator loop, a morning loop, and an evening loop (Hsu and Harmer, 2014). Key players in this interconnected network are two MYB transcription factors *CIRCADIAN CLOCK-ASSOCIATED1* (*CCA1*) and *LATE ELONGATED HYPOCOTYL* (*LHY*), and *TIMING OF CAB EXPRESSION1/PSEUDO-RESPONSE REGULATOR1* (*TOC1/PRR1*). These three components repress the activity of each other and direct temporal regulation of most other clock components (Hsu and Harmer, 2014).

The two key responses of circadian clock to high temperatures are temperature entrainment and temperature compensation (**Figure 1B**). Thermocycles are able to entrain the clock in constant light with shorter periods than photocycles in *Arabidopsis* (Boikoglou et al., 2011). The genes governing temperature entrainment remain largely unknown, except for the evening loop component *EARLY FLOWERING 3* (*ELF3*), and the morning loop components *PRR7* and *PRR9*. The etiolated *elf3-1* seedlings are unable to exhibit classic indicators of entrainment by temperature cycles in darkness (Thines and Harmon, 2010). The *prr7-3 prr9-1* double mutants fail to entrain to thermocycles of 22/12°C, but can entrain to 28/22°C thermocycles without a robust oscillation (Salome and McClung, 2005; Salome et al., 2010). Temperature compensation refers to the ability of maintaining a relatively constant period over a range of environmental temperatures. As temperature increases from 12 to 27°C, the periodicity has no significant changes in *Arabidopsis* (Gould et al., 2006). High temperature enhances the CCA1 binding affinity to the promoters of the oscillator genes, which is precisely antagonized by protein kinase CASEIN KINASE2 (CK2; Portoles and Mas, 2010) and transcription factor FLOWERING BASIC HELIX-LOOP-HELIX 1 (FBH1; Nagel et al., 2014) to maintain the circadian period. At high temperatures, the activities of CCA1 and LHY are counterbalanced by the temperature-dependent regulation of TOC1 and GI (Gould et al., 2006) as well as PRR7 and PRR9 (Salome et al., 2010). *REVEILLE8* (*RVE8*), a homolog of *CCA1* and *LHY*, is also required for temperature compensation, as *rve8* mutants have long-period and its overexpression lines have short-period phenotypes under high temperature (Rawat et al., 2011). Moreover, the activity of PRR7 is regulated by HEAT SHOCK FACTOR B2b (HsfB2b; Kolmos et al., 2014) and the evening complex night-time repressor consisting of ELF3, ELF4, and LUX ARRHYTHMO (LUX; Mizuno et al., 2014). The night-time repressor also mediate temperature responses of the clock transcriptional circuitry by regulating other targets *GI*, *LUX* and *PIF4/5*. This activity of night-time repressor is antagonized by warm temperature, suggesting that the PIF4-mediated morphological acclimation may be regulated by the night-time repressor under warm temperature (Mizuno et al., 2014). It is noteworthy that heat-induced alternative splicing of clock components such as *CCA1*, *PRR7*, *TOC1,* and *ELF3* may be an important mechanism in temperature compensation (Kwon et al., 2014; Filichkin et al., 2015). Overall, our knowledge on the response of circadian clock to high temperature is rather limited. How plants integrate circadian clock with immunity under high temperature remains elusive.

### Modulation of Plant Immunity by High Temperature

The effect of high temperatures on plant immunity has been well summarized recently (Hua, 2013). Two major influences of high temperature on plant immunity are that high temperature often inhibits the effector triggered immunity (ETI) and enhances RNA-silencing mediated resistance (**Figure 1C**). In ETI, pathogen effectors are recognized by the host proteins encoded by resistance (*R*) genes, of which most are nucleotide binding-leucine rich repeat (NB-LRR) class of proteins (Martin et al., 2003). *SUPPRESSOR OF npr1-1, CONSTITUTIVE 1* (*SNC1*) is the first identified *R* gene mediating high temperature inhibition of resistance (Yang and Hua, 2004), which is negatively regulated by *BONZAI1* (*BON1*; Zhu et al., 2010). At 22°C, the *bon1-1* loss-of-function mutation activates *SNC1*, which induces constitutive salicylic acid (SA)-mediated defense responses and inhibits plant growth. While at 28°C, the nuclear accumulation of SNC1 protein is reduced by high temperature, which may inhibit the activity of SNC1 protein and suppress the defense responses (Zhu et al., 2010). Besides *BON1*, other negative regulators of *SNC1* have been identified, such as *BON1-ASSOCIATED PROTEIN 1*(*BAP1*), *BAK1-INTERACTING RECEPTOR-LIKE KINASE 1*(*BIR1*), *SUPPRESSOR OF rps4-RLD* (*SRFR1*), *CONSTITUTIVE EXPRESSER OF PR GENES 1*(*CPR1*) and *MAP KINASE PHOSPHATASE 1* (*MKP1*; Gou and Hua, 2012). These genes tightly control SNC1 activities at both the transcriptional and posttranscriptional levels. Besides, abscisic acid (ABA) plays a positive role in the high temperature-mediated inhibition of disease resistance, as ABA deficiency promotes nuclear accumulation of SNC1 and potentiates defense responses at 28°C (Mang et al., 2012; Zhu et al., 2012b). In contrast, nitric oxide (NO) may act as a negative regulator in the high temperature-mediated inhibition of disease resistance (Wang et al., 2012a). It will be interesting to further dissect the interplay between ABA, NO, and SA-mediated defense responses under high temperature. Another major effect of high temperature in plant immunity is the enhancement of RNA-silencing mediated resistance. The underlying mechanisms will be discussed later in this review (see Small Interfering RNAs).

### Thermotolerance in Plants

The mechanisms of thermotolerance to heat stress in plants have been elaborated, including the HSFs and HSPs, ROS, phospholipids and calcium signaling pathways, and the network of hormones (**Figure 1D**; Qu et al., 2013). The thermotolerance in *Arabidopsis* consists of basal and acquired thermotolerance. The basal thermotolerance is an inherent ability for plants to survive in exposure to temperatures above the optimal for growth, while acquired thermotolerance refers to the ability to cope with lethal high temperatures after acclimatization to mild high temperatures (Clarke et al., 2004). It is reported that SA, jasmonic acid (JA) and ethylene signaling pathways and ROS scavenging are required for basal thermotolerance (Miller et al., 2008; Clarke et al., 2009). The transcriptional co-activator *MULTIPROTEIN BRIDGING FACTOR 1C* (*MBF1c*) is required for basal thermotolerance and functions upstream of SA, trehalose and ethylene signaling pathways during heat stress (Suzuki et al., 2008). Moreover, NADPH oxidases *RESPIRATORY BURST OXIDASE HOMOLOGUE* (*Rboh*) enhances the production and maintenance of ROS, which is important for basal thermotolerance (Miller et al., 2008). HSFs and HSPs play central roles in the acquired thermotolerance in plants. HSFs are the central regulators responsible for the expression of *HSP* genes. The *Arabidopsis* genome contains 21 HSF members that can be sorted into classes A, B, and C (Baniwal et al., 2004). HsfA1a is a master regulator for acquired thermotolerance that triggers the heat stress response through the induction of HsfA1b and HsfA2 expression, while HsfA2 is a major heat stress factor and induces the expression of HSPs under heat stress. HsfB1 acts as a co-regulator enhancing the activity of HsfA1a and HsfA2 (Baniwal et al., 2004). HSPs are categorized into five classes based on their approximate molecular masses: Hsp100, Hsp90, Hsp70, Hsp60, and small Hsps (sHsps). These HSPs function as molecular chaperones and play complementary and sometimes overlapping roles in stabilizing proteins and membranes and assisting in protein refolding under heat stress (Wang et al., 2004). A variety of signaling molecules, such as ABA, H_2_O_2_, ethylene, SA, calcium, and phospholipids, are also involved in acquired thermotolerance as well. These signaling molecules regulate the expression of *HSFs* and *HSPs* and protect cells against heat stress-induced oxidative damage (Song et al., 2012).

Although great progress has been achieved in the elucidation of molecular mechanisms of thermotolerance, how plants sense and transduce the signal of heat stress is still an important topic to be addressed. It is hard to define the primary heat sensor(s) as heat stress simultaneously poses a threat to almost all macromolecules and all organelles in the cells. Several putative heat sensors have been proposed, including a plasma membrane cyclic nucleotide gated calcium channel (CNGC) and two unfolded protein sensors in the endoplasmic reticulum (ER) and the cytosol (**Figure 1D**). The *CNGC2* gene in *Arabidopsis* and its ortholog *CNGCb* from *Physcomitrella patens* act as the primary heat sensors of land plant cells (Saidi et al., 2009; Finka et al., 2012). Heat shock impairs the protein stability and activates the unfolded protein response (UPR) in the ER and the cytosol. The cytosolic UPR is triggered by unfolded proteins in the cytosol and is notably regulated by HsfA2 (Sugio et al., 2009). Heat promotes the translocation of two basic leucine-zipper domain-containing transcription factors bZIP17 and bZIP28 to the nucleus. The nuclear-localized bZIPs not only activate ER chaperone genes and induce the ER-UPR, but also activate BR signaling, which is required for heat stress acclimation and growth (Che et al., 2010). Heat-induced cleavage of bZIP60 by the RNA splicing factor IRE1b also triggers the ER-UPR (Deng et al., 2011). Taken together, these results suggest that the primary heat sensor may lie in the plasma membrane, ER or cytosol. However, a heat-responsive retrograde pathway in chloroplast has recently been reported (Yu et al., 2012a). The photosynthetic apparatus in the chloroplast are the primary susceptible targets of heat stress. Through proteomic screening, the chloroplast ribosomal protein S1 (RPS1) is also identified as a heat-responsive protein. Under heat stress, RPS1 plays a critical role in modulating the translational efficiency of thylakoid proteins to maintain the stability and integrity of thylakoid membranes. The capacity of protein translation in chloroplasts generates the retrograde signals to activate HsfA2-dependent heat-responsive genes in the nucleus (Yu et al., 2012a). Thus, the chloroplasts are proposed as heat sensors as well.

## Epigenetic Regulation of Heat Responses in Plants

### DNA Methylation

DNA methylation is a biological process by which a methyl group is added to the cytosine bases of DNA to form 5-methylcytosine. In plants, DNA methylation occurs frequently in all three sequence contexts: the symmetric CG and CHG contexts (where H = A, T or C) and the asymmetric CHH context. In *Arabidopsis*, overall genome-wide levels of 24% CG, 6.7% CHG and 1.7% CHH methylation are observed (Cokus et al., 2008). DNA methylation in plants predominantly occurs on transposons and other repetitive DNA elements (Zhang et al., 2006). Different proteins are involved in the establishment, maintenance and removal of DNA methylation. *De novo* methylation in all sequence contexts is catalyzed by DOMAINS REARRANGED METHYLTRANSFERASE 2 (DRM2), and DNA methylation is maintained by three different pathways: CG methylation is maintained by METHYLTRANSFERASE 1 (MET1) and DECREASE IN DNA METHYLATION 1 (DDM1); CHG methylation is maintained by CHROMOMETHYLASE 3 (CMT3), a plant-specific DNA methyltransferase; and asymmetric CHH methylation is maintained by DRM2 (Chan et al., 2005). The DRM2 activity is regulated by the RNA-directed DNA methylation (RdDM) pathway (Matzke and Mosher, 2014). The plant-specific RNA polymerase IV (Pol IV) transcribes heterochromatic regions to generate single-stranded RNA (ssRNAs). RNA-DEPENDENT RNA POLYMERASE 2 (RDR2) then synthesizes double-stranded RNA intermediates (dsRNAs) as precursors for RNase III-class DICER-LIKE 3 (DCL3) to process into 24-nt small interfering RNAs (siRNAs). Following incorporation into ARGONAUTE 4 (AGO4), the 24-nt siRNAs base-pair with Pol V scaffold transcripts, which results in DRM2 recruitment and DNA methylation at the source loci (Matzke and Mosher, 2014). In plants, four bifunctional 5-methylcytosine glycosylases, REPRESSOR OF SILENCING 1 (ROS1), DEMETER (DME), DME-like 2 (DML2) and DML3, have been implicated in the active removal of 5-methylcytosine from DNA through the base excision repair pathway (Zhang and Zhu, 2012). DNA methylation has two main roles in plants: defending the genome against selfish DNA elements and regulating gene expression. DNA methylation induces the transcriptional gene silencing (TGS) of transgene as well as endogenous transposons and retrotransposons to maintain genome stability (Chan et al., 2005). DNA methylation of promoter regions usually inhibits transcription initiation, while methylation within the gene body quantitatively impedes transcript elongation in *Arabidopsis* (Zilberman et al., 2007).

The global methylation can be differently affected by heat in different species. Exposure of *Arabidopsis* plants to heat stress results in an increased global methylation and a higher homologous recombination frequency (HRF; Boyko et al., 2010). The up-regulation of *DRM2*, *NUCLEAR RNA POLYMERASE D 1*(*NRPD1*) and *NRPE1* in response to heat stress may contribute to the increased genome methylation in *Arabidopsis* (Naydenov et al., 2015). An increase in global methylation is also observed in Cork oak (*Quercus suber* L.) grown at 55°C (Correia et al., 2013). In *Brassica napus*, the DNA methylation levels increase more in the heat-sensitive genotype than in the heat-tolerant genotype under heat treatment (Gao et al., 2014). However, in cotton (*Gossypium hirsutum*) anthers, high temperature significantly decreases the expression of *S-ADENOSYL-L-HOMOCYSTEINE HYDROLASE1* (*SAHH1*) and DNA methyltransferases (*DRM1* and *DRM3*), resulting in the genome-wide hypomethylation at the tetrad stage and the tapetal degradation stage (Min et al., 2014). It appears that there is no consistent trend in the changes of DNA methylation under heat in different species. The methylation status of certain loci may be affected by heat stress. In developing rice seeds, the DNA methylation level of *Fertilization-Independent Endosperm1* (*OsFIE1*), a member of Polycomb Repressive Complex 2 (PRC2), is reduced and the transcript abundance of *OsCMT3* is repressed by a moderate heat stress (34°C) at 48 h after fertilization, which may lead to the misregulation of *OsFIE1* (Folsom et al., 2014).

Heat stress induces transcriptional activation of various transgenes that are previously silenced via TGS, such as the 35S promoter of *Cauliflower Mosaic Virus* and β*-glucuronidase* (*GUS*), which occurs without detectable changes in the levels of DNA methylation (Lang-Mladek et al., 2010; Pecinka et al., 2010; Tittel-Elmer et al., 2010). Warm temperature slightly increases the methylation level in some regions of *GUS* but decreases it in other regions (Zhong et al., 2013). Thus, it seems that DNA methylation is not involved in the regulation of heat responses. However, an analysis of the heat tolerance of mutants defective in DNA methylation reveals that the RdDM pathway is required for basal thermotolerance (Popova et al., 2013). Plants deficient in *NRPD2*, the second-largest subunit of Pol IV and V, are hypersensitive to heat stress, while *rdr2*, *dcl3* and *ago4* mutants are less sensitive. In *nrpd2* mutants recovering from heat, the misexpression of some protein-coding genes is associated with the epigenetic regulation of adjacent transposon remnants (transposons and retrotransposons; Popova et al., 2013). For example, the expression of the *COPIA*-like transposon *At1g29475* is induced by heat but not decreases during recovery in *nrpd2* plants, which may repress the adjacent six highly homologous auxin-responsive genes during recovery (Popova et al., 2013). Another study has also reported that the expression of *Calmodulin-like 41* (*CML41*) gene is up-regulated by high temperature with reduced DNA methylation level in the TE insertion very closely to the transcriptional start site (Naydenov et al., 2015). However, the activation of *ONSEN*, an LTR-copia type retrotransposon, could not be explained by the reduction of DNA methylation at the promoter upon heat stress (Cavrak et al., 2014). Whether the changes in DNA methylation of TEs play a causal role in the heat-induced activation of nearby genes needs to be explored.

Interestingly, in a genome-wide association analysis to detect loci with plastic response to climate, *CMT2* has been found to be associated with temperature seasonality in *Arabidopsis* (Shen et al., 2014). The accessions with *CMT2_STOP_* allele, which contains a premature stop codon, have broader geographic distribution than accessions with the wild-type allele. The *CMT2_STOP_* allele can alter the genome-wide CHH-methylation pattern and *cmt2* mutant plants have an improved heat-stress tolerance, suggesting that *CMT2*-dependent CHH methylation may act as an important alleviator of heat stress responses. Moreover, the *CMT2_STOP_* allele is associated with increased leaf serration and higher disease presence after bacterial inoculation (Shen et al., 2014). In summary, the above studies have not elaborated on the role of DNA methylation in heat responses. *cmt2* mutant plants have an improved heat-stress tolerance while *nrpd2* mutant plants are hypersensitive to heat stress, suggesting the different roles of *CMT2*-dependent CHH methylation and the RdDM pathway in response to heat (**Figure 1D**). Whether DNA methylation regulate plant immunity and circadian clock under heat needs to be investigated.

### Histone Covalent Modification

In eukaryotic cells, genomic DNA is packaged into chromatin. The fundamental unit of chromatin is the nucleosome composed of ∼147 bp- DNA wrapped around a histone octamer consisting of two copies of H2A, H2B, H3, and H4. Histone tails can be covalently modified at various amino acids and via different types, such as acetylation, mono/di/trimethylation, phosphorylation, ubiquitination, glycosylation, ADP ribosylation, carbonylation, sumoylation, and biotinylation. These modifications can activate or repress transcription by generating either ‘open’ or ‘closed’ chromatin configurations, respectively, thereby regulating the accessibility of chromatin to transcriptional regulators (Li et al., 2007). In plants, histone methylation and acetylation have been well characterized. As one of the most complex modifications, histone methylation not only occurs at distinct sites of lysine and arginine residues but also differs in the number of methyl groups added. In *Arabidopsis*, histone methylation mainly occurs at Lys4 (K4), Lys9 (K9), Lys27 (K27), Lys36 (K36), and Arg17 (R17) of histone H3, and Arg3 (R3) of histone H4 (Liu et al., 2010). These methylation types have different roles. H3K4me and H3K36me mainly generate ‘open’ chromatin configurations and activate transcription, whereas H3K9me and H3K27me create a “closed” chromatin and transcriptional repression. H3K9me2 functions as a silencing mark linked to DNA methylation, while H3K27me3 represses the expression of many genes targeted by PRC2. The effects of histone methylation on genome management, transcriptional regulation, and development in plants have been well reviewed (Liu et al., 2010; He et al., 2011). Histone acetylation and deacetylation are catalyzed by histone acetyltransferases (HATs) and histone deacetylases (HDACs), respectively. Histone acetylation are directly connected with transcriptional activation and affect a variety of biological processes in plant growth and development as well as biotic and abiotic stress responses (Chen and Tian, 2007).

Similar to DNA methylation, the histone modifications can be differently affected by heat in different species. In the unicellular green alga *Chlamydomonas reinhardtii*, there are higher levels of histone H3/4 acetylation and lower levels of H3K4me1 at promoter regions of active genes compared with inactive promoters and transcribed and intergenic regions after heat stress (Strenkert et al., 2011). The transcription factor *HSF1* may mediate the acetylation of histones H3/4, remodeling of the H3K4 methylation, and transcription initiation/elongation upon heat stress (Strenkert et al., 2011). However, temperature shift from 25 to 45°C decreases the acetylated histone H3 levels in the forest tree Cork oak (Correia et al., 2013). The deacetylated H3 may be responsible for repressive chromatin in gene promoters and repression of gene transcription. Histone modifications are also involved in rice seed and cotton anther development at high temperature. The H3K9me2 level of *OsFIE1* is sensitive to moderate heat stress and may be an important component involved in regulating *OsFIE1* when developing rice seeds are exposed to a moderate heat stress (Folsom et al., 2014). In cotton anthers, one histone methyltransferase, one histone monoubiquitination gene and two jumonji C (jmjC) domain-containing genes are down-regulated upon high temperature (Min et al., 2014). The roles of the differently regulated histone modifications in heat responses remain unknown.

After heat stress, the levels of H3K9me2, H3K27me1 and H3K4me3 at a transcriptionally silenced GUS transgene (TS-GUS) and a non-LTR retrotransposon LINE039 showed only minor changes or remain unchanged (Lang-Mladek et al., 2010). But the amounts of H3K9ac1 and H3K9/14ac2 significantly increased in response to heat. The histone deacetylase HDA6 may be involved in this process as the TS-GUS activity showed a pronounced increase in *hda6* mutants (Lang-Mladek et al., 2010). Similarly, another study also demonstrates that levels of H3K4me3, H3K9me2, H3K27me2, and H3K27me3 were unaffected by temperature shift from 4 to 37°C for 15 h while a modest enrichment in H3K9ac-K14ac was detected at 5S rDNA, 106B long terminal-like dispersed repeats and a *Mutator*-like transposable element related locus MULE-F19G14 (Tittel-Elmer et al., 2010). Thus, the heat-induced release of silencing seems to be associated with histone acetylation but not histone methylation. However, similar to the wild-type control, the transcripts from these three targets over-accumulated in *hda6* mutants exposed to temperature shift, but reverted to the initial level after 2 days of recovery. These results exclude the possibility that HDA6 activity is required for the release of gene silencing (Tittel-Elmer et al., 2010). Interestingly, both the levels of repressive H3K9me2 and active H3K4me3 significantly reduced directly after long heat stress (37°C for 30 h) and returned to the initial level after 7 days of recovery (Pecinka et al., 2010). After long heat stress, nucleosomes and all the histone modifications on them were partially removed through unknown mechanisms, and then reloaded to the chromatin upon returned to ambient temperatures, while the levels of histone modifications on the remaining histones remained relatively unchanged (Pecinka et al., 2010). Thus, histone modifications may not play an important role in the heat-induced release of silencing.

Recently, environmental history of repetitive heat stress has been reported to modulate *Arabidopsis* pattern-triggered immunity (PTI) in a HISTONE ACETYLTRANSFERASE1 (HAC1)-dependent manner (**Figure 1D**; Singh et al., 2014). *Arabidopsis* plants exposed to repetitive heat stress were more resistant to virulent bacteria than plants grown in a more stable environment. The enhanced resistance in repetitively stress-challenged plants occurred with priming of PTI-responsive genes and the potentiation of PTI-mediated callose deposition. The transcriptional activation of PTI-responsive genes was associated with enrichment of H3K9/14ac, H3K4me2 and H3K4me3, indicating a positive relationship between the bacterial resistance and histone modifications after heat stress. In *hac1-1* mutants, repetitively heat stress failed to induce enhanced resistance to bacteria, priming of PTI, and enrichment of H3K9/14ac, H3K4me2 and H3K4me3. These findings reveal that HAC1 is a necessary component for bacterial resistance, priming of PTI, and open chromatin configurations mediated by repetitive heat stress exposure. Whether H3K4 methylation have a similar role needs to be further analyzed (Singh et al., 2014). Multiple histone modifications, such as H3K56ac, H3K9/14ac, H3K4me3, and H3K4me2, have been found to closely correlate with the rhythmic expression of *LHY*, *CCA1*, *TOC1*, *PRR7*, and *PRR9* in *Arabidopsis* (Seo and Mas, 2014). Histone acetylation may contribute to the circadian peak of expression by influencing transcription factor accessibility under different temperature conditions, while H3K4me3 may antagonize clock repressor binding, ensuring a proper timing and duration of gene activation (**Figure 1B**). Overall, the roles of histone modifications in response to heat stress are largely obscure and need to be further recognized.

## Histone Chaperones

Histone chaperones are a group of proteins that bind histones and prevent non-productive aggregation between highly positive charged histones and highly negative charged DNA without using the energy of ATP (Zhu et al., 2012a). They play a crucial role in nucleosome assembly during different processes such as DNA replication, repair, and transcription. In general, histone chaperones can be classified as either H3–H4 or H2A–H2B chaperones on the basis of their preferential histone binding. In plants, the well-studied chaperones include H3–H4 chaperones ANTI-SILENCING FUNCTION 1 (ASF1), CHROMATIN ASSEMBLY FACTOR-1 (CAF-1) and HISTONE REGULATORY HOMOLOG A (HIRA), and the H2A–H2B chaperones NUCLEOSOME ASSEMBLY PROTEIN1 (NAP1), NAP1-RELATED PROTEIN (NRP) and FACILITATES CHROMATIN TRANSCRIPTION (FACT; Zhu et al., 2012a). Only a few studies have reported the role of histone chaperones in heat responses. The reload of nucleosome, whose occupancy is reduced by long heat stress, requires the CAF-1-dependent chromatin assembly complex (Pecinka et al., 2010). Wild-type plants lost nucleosomes immediately after heat stress and restored the original level during recovery. By contrast, the *fasciata1* (*fas1*) and *fas2* mutants that lack different subunits of CAF-1, had the already reduced nucleosome occupancy before heat treatment. The nucleosome occupancy was further reduced by long heat stress in the mutants, and there was no restoration even after 7 days of recovery. The heat stress-induced loss of nucleosomes and heterochromatin decondensation led to the activation of transcriptionally silenced repetitive elements. The CAF-1-dependent chromatin assembly complex may provide a safeguarding mechanism to minimize the heat-induced epigenetic damage in the germ line (Pecinka et al., 2010). *AtASF1A* and *AtASF1B* have also been reported to participate in basal and acquired thermotolerance (**Figure 1D**; Weng et al., 2014). Upon heat stress, AtASF1A/B proteins were recruited onto chromatin, and their enrichment was correlated with nucleosome removal and RNA polymerase II accumulation at the promoter and coding regions of some *HSF* and *HSP* genes. Moreover, AtASF1A/B facilitated H3K56ac, which also promotes the activation of some *HSFs* and *HSPs* (Weng et al., 2014).

### Histone Variants

In addition to the conventional histones, which are deposited mostly during the S phase of the cell cycle, all eukaryotes have non-allelic histone variants that can be incorporated into nucleosomes in a DNA replication-independent manner during the entire cell cycle. Histone variants can alter the properties of the nucleosomes they occupy and play important roles in maintenance of genome stability, transcriptional activation and repression (Kamakaka and Biggins, 2005). There are 15 histone H3 genes in the *Arabidopsis* genome, including six canonical H3.1 or H3.1-like genes, eight H3.3 or H3.3-like genes and one centromeric histone H3 gene (Okada et al., 2005). The roles of H3 variants in heat responses have not been reported hitherto. Thirteen H2A-encoding genes have been identified in *Arabidopsis*, including four canonical H2A genes, two H2A.X genes, three H2A.Z genes and other four less categorized genes (March-Diaz and Reyes, 2009). Recently, an important study revealed that H2A.Z-containing nucleosomes mediate the thermosensory response in *Arabidopsis* (Kumar and Wigge, 2010). In a genetic screen of mutants defective in heat sensing, the *ARP6* gene was identified to mediate the response to increased temperature. The APR6 protein is an essential component of the SWR1 complex required for H2A.Z incorporation into chromatin (March-Diaz and Reyes, 2009). When grown at 22°C, the *arp6* mutants display phenotypes similar to wild-type plants grown at 27°C, such as hypocotyl and petiole elongation, leaf hyponasty, and early flowering. It is proposed that H2A.Z occupancy represses gene expression by creating a physical block to transcription or by preventing the binding of transcription activators at cooler temperatures, and eviction of H2A.Z at higher temperatures would thereby facilitate transcription of target genes. This temperature-induced H2A.Z nucleosome dynamics has been proved to regulate the binding of PIF4 to the FT promoter, thereby controlling the thermosensory activation of flowering. Based on these results, H2A.Z-containing nucleosomes are recognized as temperature sensors in the nucleus (**Figure 1A**; Kumar et al., 2012). However, it is unclear whether this mechanism is also responsible for the regulation of other heat-induced genes, such as auxin biosynthesis genes required for warm temperature-mediated morphological acclimation, *HSF* and *HSP* genes in acquired thermotolerance, and genes involved in the modulation of plant immunity and circadian clock by high temperature.

### ATP-Dependent Chromatin Remodeling

ATP-dependent chromatin remodeling complexes use the energy of ATP hydrolysis to alter the structure of chromatin by destabilizing histone–DNA interactions, moving histone octamers or catalyzing the incorporation of histone variants. According to ATPases used, the complexes can be grouped into four main classes: the SWItch/Sucrose Non-Fermentable (SWI/SNF) class, the imitation switch (ISWI) class, the inositol requiring 80 (INO80) class, and the chromodomain and helicase-like domain (CHD) class (Clapier and Cairns, 2009). The SWI/SNF complex is the first ATP-dependent chromatin remodeling complex identified, and 41 SNF2 proteins in *Arabidopsis* have yet been identified. Functional analysis indicates that many of these proteins play important roles in plant development and stress response. Moreover, some of these proteins are involved in epigenetic regulation, such as TGS (dependent or independent of DNA methylation), H2A.Z deposition and histone modifications (Knizewski et al., 2008).

The Swi2/Snf2-related (SWR1) complex regulates transcription by replacing the H2A–H2B histone dimers in nucleosome with dimers containing the H2A.Z variant. As mentioned above, the ARP6 protein, which is an essential component of the SWR1 complex, plays an important role in temperature sensing (**Figure 1A**; Kumar and Wigge, 2010). The SNF2/Brahma-type chromatin-remodeling gene *CHROMATIN REMODELING* (*CHR12*) also plays a vital role in mediating the temporary growth arrest of *Arabidopsis* under heat, drought and salinity stresses (**Figure 1D**; Mlynarova et al., 2007). When exposed to stress conditions, a gain-of-function mutant overexpressing *AtCHR12* showed growth arrest of normally active primary buds and reduced growth of the primary stem. In contrast, the loss-of-function mutant showed less growth arrest than the wild-type when exposed to moderate stress (Mlynarova et al., 2007). In *Chlamydomonas reinhardtii*, heat stress induces low nucleosome occupancy at promoter regions of active genes, which is mediated by HSF1 and other unknown chromatin remodeling complexes (Strenkert et al., 2011). However, the heat stress-mediated release of TGS is at least partly independent of the activity of MORPHEUS’ MOLECULE 1 (MOM1)/CHR15, a well-known DNA methylation-independent transcriptional silencer, and DECREASED DNA METHYLATION 1 (DDM1)/CHR1, a component required for DNA methylation and H3K9me2 (Tittel-Elmer et al., 2010).Whether other ATP-dependent chromatin remodeling complexes play roles in heat responses remains elusive.

### Small RNAs

Small RNAs are 18–30 nt non-protein-coding RNAs, which have emerged as key guide molecules in the control of gene expression. Two major types of small RNAs in plants, microRNAs (miRNAs) and siRNAs, are distinguished by the different proteins involved in their biogenesis and the modes of regulation (Ghildiyal and Zamore, 2009).

#### microRNAs

Plant miRNAs are a class of 20–24 nt endogenous small RNAs that derive from the miRNA genes (*MIR*; Rogers and Chen, 2013). *MIR* genes are transcribed by Pol II to generate primary miRNA transcripts called pri-miRNAs. The pri-miRNAs are processed into stem–loop precursor pre-miRNA and further excised as miRNA/miRNA^∗^ duplex by the endonuclease activity of the DCL1 protein complex in the nucleus. The mature miRNAs are exported to the cytoplasm and incorporated into AGO proteins, mediating posttranscriptional gene silencing (PTGS) through slicing or translational inhibition, or TGS by targeting chromatin for cytosine methylation (Rogers and Chen, 2013).

A diversity of conserved and non-conserved heat-responsive miRNAs have been identified by small RNA deep-sequencing in different species, but few of them have been validated by either northern blots or real time PCR. As listed in **Table 2**, most of the conserved heat-responsive miRNAs are differently regulated in various species, except for miR159, 166 and 472 families. *miR159* has been found to be down-regulated by heat in *Arabidopsis* (Zhong et al., 2013), wheat (*Triticum aestivum*; Wang et al., 2012b; Kumar et al., 2014) and cassava (*Manihot esculenta*; Ballen-Taborda et al., 2013). The main targets of *miR159* are *MYB* transcription factors. *Tae-miR159* has been demonstrated to direct the cleavage of *TaGAMYB1* and *TaGAMYB2* (Wang et al., 2012b). The *tae-miR159* overexpression rice lines and *Arabidopsis myb33myb65* double mutants are more sensitive to heat stress relative to the wild-types, indicating that the down-regulation of *miR159* and up-regulation of its targets after heat stress might participate in a heat stress-related signaling pathway and contribute to heat stress tolerance (Wang et al., 2012b). *MiR166*, which targets HD-Zip transcription factors, is up-regulated by heat in *Arabidopsis* (Zhong et al., 2013), wheat (Xin et al., 2010), and barley (*Hordeum vulgare*; Kruszka et al., 2014). The heat-induced up-regulation of *hvu-miR166a* and down-regulation of its targets, *PHAVOLUTA* (*PHV*), *REVOLUTA* (*REV*) and a homeobox-leucine zipper protein HOX9-like gene, might influence the leaf morphology (Kruszka et al., 2014). *miR472* may be down-regulated by heat in *Arabidopsis* (Zhong et al., 2013) and Chinese white poplar (*Populus tomentosa*; Chen et al., 2012), but need to be further validated.

**Table 2:** The conserved heat-responsive miRNAs in different plant species.

Family	*miRNA*^a^	Heat treatment	Regulation	Validation^c^	Target proteins
156	*ath-miR156h*	AT^b^	Up-regulation	Yes	SPL transcription factor
	*ath-miR156g,h*	Grown at 30°C	Up-regulation	No	
	*bra-miR156g,h*	46°C for 1 h	Up-regulation	Yes	BracSPL2
	*osa-miR156a,g,h*	42°C day/36°C night for 24 h	Down-regulation in roots and shoots	Yes	SPL transcription factor
	*tae-miR156a-g*	40°C for 2 h	Up-regulation	Yes	SPL transcription factor
	*tae-miR156*	42°C for 2 h	Up-regulation	Yes	Heat shock protein 90
	*mes-miR156a*	37°C for 24 h	Down-regulation	Yes	SPL transcription factor
159	*ath-miR159a,b*	Grown at 30°C	Down-regulation	No	MYB and TCP transcription factors
	*tae-miR159*	40°C for 2 h	Down-regulation	Yes	GAMYB1 and GAMYB2
	*tae-miR159a,b*	42°C for 2 h	Down-regulation	Yes	WRKY transcription factor; MYB3; alkaline phosphatase family protein, cytochrome P450, cobalamine adenosyl transferase, Mob1-like protein and TLD family protein
	*mes-miR159a*	37°C for 24 h	Down-regulation	Yes	myb-like HTH transcriptional regulators
160	*ath-miR160a-c*	Grown at 30°C	Up-regulation	No	Auxin response factors
	*osa-miR160a*	42°C day/36°C night for 24 h	Down-regulation in roots, up-regulation in shoots	Yes	
	*pto-miR160a-d*	37°C for 8 h	Down-regulation	No	
	*tae-miR160*	40°C for 2 h	Up-regulation	No	Heat shock protein 70; ARF; tetratricopeptide repeat (TPR)
	*tae-miR160*	42°C for 2 h	Down-regulation	Yes	
	*hvu-miR160a*	35.5°C for 24 h	Up-regulation	Yes	ARF17 and ARF13
	*mes-miR160a*	37°C for 24 h	Down-regulation	Yes	Auxin response factor
	*celery-miR160*	38°C for 1 h	Up-regulation	Yes	Auxin response factor
162	*osa-miR162a*	42°C day/36°C night for 24 h	Down-regulation in both roots and shoots	Yes	Endoribonuclease DCL1
164	*ath-miR164a-c*	Grown at 30°C	Up-regulation	No	NAC domain containing transcription factors
	*tae-miR164*	42°C for 2 h	Down-regulation	Yes	Small heat shock proteins 17; NAC transcription factor; target genes involved in mitogen-activated protein kinase (MAPK) signaling pathways
	*celery-miR164*	38°C for 1 h	Up-regulation	Yes	NAC domain containing transcription factors
166	*ath-miR166a*	Grown at 30°C	Up-regulation	No	HD-Zip transcription factors including PHV and REVOLUTA
	*tae-miR166a-d*	40°C for 2 h	Up-regulation	Yes	Unknown
	*hvu-miR166a*	35.5°C for 24 h	Up-regulation	Yes	HD-Zip transcription factors including PHV and REVOLUTA; a homeoboxleucine zipper protein HOX9-like gene
167	*ath-miR167c,d*	Grown at 30°C	Up-regulation	No	HD-Zip transcription factors including PHV and PHB
	*ath-miR167d*	AT^b^	Down-regulation	No	
	*bra-miR167*	42°C for 3 h per day for 7 days	Up-regulation	No	TOM1-like protein 2; Tudor domain-containing protein 3
	*bra-miR167^∗^*	42°C for 3 h per day for 7 days	Up-regulation	No	GDSL esterase/lipase; Ribulose bisphosphate carboxylase/oxygenase activase
	*bra-miR167*	46°C for 1 h	Up-regulation	No	BracARF6
	*osa-miR167a,c,d*	42°C day/36°C night for 24 h	Down-regulation in both roots and shoots	Yes	Class III HD-Zip protein 4; heat repeat family protein
	*sja-miR167a*	20°C for 3h	Down-regulation	No	Unknown
	*pto-miR167c,d,f,g*	37°C for 8 h	Up-regulation	Yes	Unknown
	*tae-miR167*	42°C for 2 h	Up-regulation	Yes	Dnaj heat shock n-terminal domain-containing protein
	*hvu-miR167h*	35.5°C for 24 h	Up-regulation	Yes	ARF8 and a serine/threonine-protein kinase Nek5-like gene
168	*bra-miR168*	42°C for 3h per day for 7 days	Up-regulation	Yes	BraAGO1
	*osa-miR168a*	42°C day /36°C night for 24 h	Down-regulation in shoots, no expression in roots	Yes	AGO1
	*sja-miR168a*	20°C for 3 h	Down-regulation	No	Unknown
	*pto-miR168a,b*	37°C for 8 h	Down-regulation	Yes	Unknown
	*tae-miR168*	40°C for 2 h	Up-regulation	No	Unknown
	*celery-miR168*	38°C for 1 h	Up-regulation	Yes	Unknown
169	*ath-miR169a,d-n*	Grown at 30°C	Down-regulation	No	CCAAT Binding Factor (CBF) and HAP2-like transcription factors
	*ath-miR169d,e,k,i,m*	AT^b^	Down-regulation	No	
	*ath-miR169b,c*	Grown at 30°C	Up-regulation	No	
	*osa-miR169a,b,g*	42°C day/36°C night for 24 h	Down-regulation in roots, up-regulation in shoots	Yes	Nuclear transcription factor Y subunit
	*pto-miR169j-m*	37°C for 8 h	Up-regulation	No	Unknown
	*pto-miR169n-t*	37°C for 8 h	Down-regulation	No	Unknown
	*tae-miR169a-d*	40°C for 2 h	Up-regulation	No	Unknown
171	*ath-miR171a-c*	Grown at 30°C	Up-regulation	No	Scarecrow-like transcription factors
	*ath-miR171b,c*	AT^b^	Up-regulation	No	
	*bra-miR171a-1*	42°C for 3 h per day for 7 days	Up-regulation	No	26S protease regulatory subunit 6A homolog
	*pto-miR171a-i*	37°C for 8 h	Down-regulation	No	Unknown
	*tae-miR171a*	42°C for 2 h	Down-regulation	Yes	Scarecrow-like protein
	*ptc-miR171l-n*	37°C for 24 h	Down-regulation	Yes	SCL, clathrin assembly protein
172	*ath-miR172b^∗^*	Grown at 30°C	Down-regulation	No	Eukaryotic translation initiation factor 5, putative; calcium-transporting ATPase
	*ath-miR172c,d,e*	Grown at 30°C	Up-regulation	No	AP2 transcription factors
	*ath-miR172a,e*	AT^b^	Up-regulation	No	
	*tae-miR172a,b*	40°C for 2 h	Down-regulation	Yes	AP2 transcription factors; floral homeotic protein
319	*ath-miR319a,b*	Grown at 30°C	Up-regulation	No	MYB and TCP transcription factors
	*ath-miR319a,c*	AT^b^	Up-regulation	No	
	*ath-miR319c*	Grown at 30°C	Down-regulation	No	
	*tae-miR319*	42°C for 2 h	Down-regulation	Yes	MYB3; histone protein-associated genes
393	*ath-miR393a,b*	Grown at 30°C	Down-regulation	No	F-box proteins and bHLH transcription factors
	*tae-miR393*	40°C for 2 h	Up-regulation	Yes	Genes involved in auxin signaling pathway and basal defense
394	*ath-miR394a,b*	Grown at 30°C	Down-regulation	No	F-box proteins
	*celery-miR394*	38°C for 1 h	Up-regulation	Yes	Unknown
	*pto-miR394a,b*	37°C for 8 h	Down-regulation	No	Unknown
395	*ath-miR395a-f*	Grown at 30°C	Up-regulation	No	ATP sulphurylases; leucine-rich repeat family protein
	*ath-miR395d*	AT^b^	Up-regulation	No	
	*pto-miR395a-j*	37°C for 8 h	Down-regulation	Yes	Unknown
	*sja-miR395x*	20°C for 3 h	Up-regulation	No	Unknown
	*tae-miR395b*	42°C for 2 h	Up-regulation	No	sulfur transporters and ATP sulphurylases
	*celery-miR395*	38°C for 1 h	Up-regulation	Yes	Unknown
396	*ath-miR396a*	Grown at 30°C	Up-regulation	No	Growth-regulating factor (GRF) transcription factors; rhodenase-like proteins; kinesin-like protein B
	*han-miR396*	37°C for 24 h	Down-regulation	Yes	HaWRKY6
397	*ath-miR397a,b*	Grown at 30°C	Up-regulation	No	Laccases and beta-6 tubulin
	*osa-miR397b.2*	42°C for 8 h	Up-regulation	Yes	L-ascorbate oxidase
	*osa-miR397*	42°C day/36°C night for 24 h	Down-regulation in shoots, no epression in roots	Yes	L-ascorbate oxidase precursor; F-box domain containing protein
	*mes-miR397a*	37°C for 24 h	Down-regulation	Yes	Laccase/Diphenol oxidase family protein
398	*ath-miR398b*	Grown at 30°C	Up-regulation	No	CSD1, CSD2 and CCS
	*ath-miR398*	37°C for 4 h	Up-regulation	Yes	
	*bra-miR398a,b*	46°C for 1 h	Down-regulation	Yes	BracCSD1
	*osa-miR398*	42°C day/36°C night for 24 h	Down-regulation in both roots and shoots	Yes	Superoxide dismutase (SOD) gene family
	*sja-miR398a-5p*	20°C for 3 h	Up-regulation	No	Unknown
	*tae-miR398*	42°C for 2 h	Up-regulation	Yes	Superoxide dismutase (SOD) gene family
399	*ath-miR399b-d, f*	Grown at 30°C	Up-regulation	No	Phosphatase transporter
	*ath-miR399c,d*	AT^b^	Down-regulation	No	
	*bra-miR399b*	46°C for 1 h	Down-regulation	No	BracPHO2
400	*ath-miR400*	37°C for 12 h	Down-regulation	Yes	Pentatricopeptide (PPR) repeat-containing protein
408	*ath-miR408*	Grown at 30°C	Up-regulation	No	Peptide chain release factor; plantacyanin
	*sja-miR408b-5p*	20°C for 3 h	Down-regulation	Yes	An unknown conserved protein
	*pto-miR408*	37°C for 8 h	Down-regulation	Yes	Unknown
	*mes-miR408*	37°C for 24 h	Down-regulation	Yes	Plantacyanin
	*celery-miR408*	38°C for 1 h	Up-regulation	Yes	Unknown
472	*ath-miR472*	Grown at 30°C	Down-regulation	No	RFL1 (RPS5-LIKE 1)
	*pto-miR472a,b*	37°C for 8 h	Down-regulation	No	Disease resistance protein; F-box protein
482	*pto-miR482*	37°C for 8 h	Down-regulation	Yes	Unknown
827	*ath-miR827*	Grown at 30°C	Down-regulation	No	SPX (SYG1/Pho81/XPR1) domain-containing protein; DNA-binding storekeeper protein-related
	*ath-miR827*	AT^b^	Down-regulation	No	
	*bra-miR827*	46°C for 1 h	Down-regulation	Yes	Unknown
	*tae-miR827*	40°C for 2 h	Up-regulation	No	Unknown
	*ptc-miR827*	37°C for 24 h	Down-regulation	Yes	Sec14 cytosolic factor family protein
1117	*tae-miR1117*	42°C for 2 h	Down-regulation	Yes	Calcium dependent protein kinase 1
1450	*pto-miR1450*	37°C for 8 h	Up-regulation	No	Unknown
	*ptc-miR1450*	37°C for 24 h	Down-regulation	Yes	Leucine-rich repeat transmembrane protein kinase

*The miRNA family, kinds of heat treatment, up or down-regulation, validated by either northern blot or realtime PCR, and their target proteins are presented. ^a^ath: *Arabidopsis thaliana* (Yan et al., 2012; Guan et al., 2013; Zhong et al., 2013; Stief et al., 2014); bra: *Brassica rapa* (Yu et al., 2012b; Bilichak et al., 2015); celery: *Apium graveolens* (Li et al., 2014a); han:*Helianthus annuus* (Giacomelli et al., 2012); hvu: *Hordeum vulgare* (Kruszka et al., 2014); mes: *Manihot esculenta* (Ballen-Taborda et al., 2013); osa: *Oryza sativa* (Jeong et al., 2011; Sailaja et al., 2014); ptc: *Populus trichocarpa* (Lu et al., 2008); pto: *P. tomentosa* (Chen et al., 2012); sja: *Saccharina japonica* (Liu et al., 2014); tae: *Triticum aestivum* (Xin et al., 2010; Wang et al., 2012b; Kumar et al., 2014). ^b^AT: acquired thermotolerance, pretreated at 37°C for 1 h and returned to 22°C for 1.5 h for recovery, treated at 44°C for 45 min. ^c^validated by either northern blot or realtime PCR*.

Several miRNA families seem to be responsive to heat in at least four species, including miR156, 160, 167, 168, 169, 171, 395, 398, 408, and 827 families (**Table 2**). Some members of the miR156 family are induced by heat in *Arabidopsis* (Zhong et al., 2013; Stief et al., 2014), *Brassica rapa* (Yu et al., 2012b), and wheat (Xin et al., 2010; Kumar et al., 2014), but are repressed by heat in rice (Sailaja et al., 2014) and cassava (Ballen-Taborda et al., 2013). In *Arabidopsis*, *miR156* isoforms are highly induced after heat stress and target *SQUAMOSA-PROMOTER BINDING-LIKE* (*SPL*) transcription factor genes (especially *SPL2* and *SPL11*) that are master regulators of developmental transitions (Stief et al., 2014). AGO1 acting through *miR156* and its target *SPLs* appears to mediate the adaptation to recurring heat stress (HS memory) by inducing the expression of HS memory–related genes (Stief et al., 2014). *Bra-miR156h* and *bra-miR156g* were also heat-induced and their putative target *BracSPL2* was down-regulated (Yu et al., 2012b). The up-regulation of *tae-miR156* and down-regulation of its putative target *SPL* genes *Ta3711* and *Ta7012* were also validated in wheat (Xin et al., 2010). However, the roles of the down-regulated *miR156* in rice (Sailaja et al., 2014) and cassava (Ballen-Taborda et al., 2013) remain unknown. The regulation of miR160 family by heat is quite different in various species, although they all target auxin response factors (ARFs). After heat stress, *miR160* was up-regulated in *Arabidopsis* (Zhong et al., 2013), barley (Kruszka et al., 2014) and celery (*Apium graveolens*; Li et al., 2014a), but down-regulated in cassava (Ballen-Taborda et al., 2013) and Chinese white poplar (Chen et al., 2012). An increase in barley *miR160a* during heat stress down-regulated the expression level of *ARF17* and *ARF13*, which might affect shoot morphology and root growth (Kruszka et al., 2014). *miR160* as well as *miR169* in rice showed differential expression in roots and shoots under heat stress, suggesting the different regulation of the target genes by heat in this two different tissues (Sailaja et al., 2014). It is also amazing that *miR160* in wheat was up-regulated by 40°C for 2 h in the heat tolerant genotype TAM107 (Xin et al., 2010), but down-regulated with the up-regulation of its putative target *HSP70* by 42°C for 2 h in another heat tolerant cultivar HD2985 (Kumar et al., 2014). *miR167* was proved to be up-regulated in Chinese white poplar (Chen et al., 2012), wheat (Kumar et al., 2014) and barley (Kruszka et al., 2014) but down-regulated in rice (Sailaja et al., 2014). Heat stress enhanced the *miR167h*-guided cleavage of the *ARF8* and *NEK5* transcript in barley (Kruszka et al., 2014). *miR168* has also been shown to be up-regulated by heat in *Brassica rapa* (Bilichak et al., 2015) and celery (Li et al., 2014a), but down-regulated in Chinese white poplar (Chen et al., 2012) and rice (Sailaja et al., 2014). A differential expression of *bra-miR168* following heat shock in the parental tissues was observed to be negatively correlated with transcript levels of its putative target *braAGO1* in the corresponding tissues, suggesting the important role of *bra-miR168* in heat responses (Bilichak et al., 2015).

The miR398 family have been validated to be up-regulated in *Arabidopsis* (Guan et al., 2013) and wheat (Kumar et al., 2014), but down-regulated in rice (Sailaja et al., 2014) and *Brassica rapa* (Yu et al., 2012b). Heat stress rapidly induced *ath-miR398* and reduced transcripts of its target genes *COPPER/ZINC SUPEROXIDE DISMUTASE 1*(*CSD1*), *CSD2* and *COPPER CHAPERONE FOR SOD 1* (*CCS*) that control ROS accumulation (Guan et al., 2013). The altered redox status contributed to the consequent accumulation of HSFs and HSPs that are critical for thermotolerance. Transgenic plants overexpressing *miR398*-resistant versions of *CSD1*, *CSD2,* or *CCS* under the control of their native promoters were hypersensitive to heat stress, and the expression of many HSF and HSP genes under heat stress was reduced in these plants. In contrast, *csd1*, *csd2,* and *ccs* plants were more tolerant to heat stress than wild-type plants with the increased expression levels of HSF and HSP genes. Moreover, HSFA1b and HSFA7b were found to be responsible for heat induction of *miR398*. Thus, *HSFs*, *miR398* and its target genes *CSD1*, *CSD2,* and *CCS* form an essential regulatory loop for thermotolerance in *Arabidopsis* (Guan et al., 2013). However, in *Brassica rapa*, heat stress reduced the expression of the conserved miRNAs *bra-miR398a* and *bra-miR398b*, which guides heat response of their target gene *BracCSD1* (Yu et al., 2012b).The expression of most members in miR169, 171, 395, and 827 families have not been experimentally validated, and their targets remain largely unknown (**Table 2**). In addition to the above miRNA families, a lot of conserved miRNA families response to heat only in 1–3 species (**Table 2**). Among them, *han-miR396* in sunflower (*Helianthus annuus*) was found to be repressed by high temperature, which results in the up-regulation of the putative target *HaWRKY6* (Giacomelli et al., 2012). But plants overexpressing *miR396*-resistant versions of *HaWRKY6* were hypersensitive to heat shock, indicating that *HaWRKY6* is involved in a fine modulation in response to heat (Giacomelli et al., 2012).

In addition to the conserved miRNAs, many non-conserved and novel heat-responsive miRNAs have been validated. For instance, *ptc-miR1445, 1446a-e* and *1447* were down-regulated by heat in *P. trichocarpa* (Lu et al., 2008); *pto-smR7*, *8,* and *9* were down-regulated by heat in *P. tomentosa* (Chen et al., 2012); *osa-miR1884* was down-regulated in roots but up-regulated in shoots by heat (Sailaja et al., 2014); *tae-candidate_3466* and *5064* were up-regulated in wheat by heat (Kumar et al., 2014). Interestingly, the splicing of introns hosting *miR160a* and *miR5175a* in barley was heat induced, but the roles of these spliced isoforms in response to heat stress are unclear (Kruszka et al., 2014). Such heat stress-induced alternative splicing also regulates the *miR400* expression in *Arabidopsis* (Yan et al., 2012). The intronic *MIR400* is co-transcribed with its host gene *At1g32583*. Upon heat stress, a specific alternative splicing occurred at the first intron of *At1g32583* containing the *miR400* hairpin, which led to a decrease of mature *miR400*, but did not affect the host gene expression. This alternative splicing event may be favorable for thermotolerance, as overexpression of *MIR400* made the plants more sensitive to heat stress (Yan et al., 2012). These findings extend our view about the regulatory mechanism linking miRNAs and heat stress.

It is worth noting that some heat-responsive miRNAs also function in other biotic and abiotic stresses. The *miR156-SPL* pathway in rice also functions in other stresses such as cold, salt and drought stress, suggesting a vital role of *miR156* in modulating plant development and responses to abiotic stress (Cui et al., 2014). *tae-miR827* and *2005* were up-regulated in wheat by both powdery mildew infection and heat stress (Xin et al., 2010). *ptc-miR 171l-n*, *530a*, *1445*, *1446a-e,* and *1447* were down-regulated in response to heat as well as cold, salt and dehydration in *P. trichocarpa* (Lu et al., 2008). *mes-miR156a*, *159a*, *160a*, *397a,* and *408* were down-regulated by heat and drought stresses in cassava (Ballen-Taborda et al., 2013). Thus, miRNAs may integrate the regulatory networks of heat stress with that of other biotic and abiotic stresses.

#### Small Interfering RNAs

Plant siRNAs are processed by DCL2-4 from long dsRNAs, which are generated directly from virus replication and inverted repeats (IRs), or converted from ssRNAs by RDRs, or by annealing of two complementary and separately transcribed RNA strands (Bologna and Voinnet, 2014). SiRNAs guide a silencing effector complex to homologous DNA loci to trigger TGS or target mRNAs for transcript cleavage. Several exogenously triggered PTGS pathways resulting in transcript cleavage have been reported. These PTGS pathways can be induced by sense transgenes (S-PTGS), antisense transgenes (A-PTGS), inverted-repeat transgenes (IR-PTGS) and virus replication (VIGS). The diverse PTGS pathways play important roles in plant immunity and silencing of transgenes (Brodersen and Voinnet, 2006). In *Arabidopsis*, several distinct classes of endogenous siRNAs have also been uncovered, including repeat-associated siRNAs (ra-siRNAs), trans-acting siRNAs (ta-siRNAs), natural antisense transcript-derived siRNAs (nat-siRNAs), endogenous IR-derived siRNAs, and double-strand-break-induced RNAs (diRNAs; Bologna and Voinnet, 2014). Ra-siRNAs are typically 24-nt small RNAs that are derived from genomic repetitive sequences, which usually direct DNA methylation through the RdDM pathway (Matzke and Mosher, 2014). The ta-siRNAs arise from eight recognized *Arabidopsis TAS* loci (*TAS1a-c*, *TAS2*, *TAS3a-c*, and *TAS4*) through a miRNA-dependent biogenesis pathway (Fei et al., 2013). Ta-siRNAs are distinguished for the ability to function in *trans* to suppress the expression of target genes, such as disease resistance genes and transcription factors. Nat-siRNAs, which originate from the overlapping region of a pair of natural antisense transcripts (NAT), have been found a role in stress responses. In the case of two published nat-siRNAs, one transcript of the NAT pair is constitutively expressed and the other is induced by salt or bacterial pathogen, which induce the production of nat-siRNAs (Borsani et al., 2005; Katiyar-Agarwal et al., 2006). Nat-siRNAs target the constitutive expressed transcript for cleavage, which confers tolerance to the inductive stress. Endogenous IR-derived siRNAs are processed by DCLs from genomic loci rearranged to form extended IRs that produce perfect or near-perfect dsRNA molecules (Dunoyer et al., 2010). IR-derived siRNAs can drive non-cell-autonomous silencing at both transcriptional and posttranscriptional levels and may have adaptive value by integrating temporally and/or spatially restricted stresses or environmental signals at the whole-plant level and perhaps in progenies (Dunoyer et al., 2010). DiRNAs derive from both sense and antisense strands around double-strand-break sites and may function as guide molecules directing chromatin modifications or the recruitment of protein complexes to source sites to facilitate repair (Wei et al., 2012).

In addition to miRNAs, the diverse exogenous and endogenous siRNAs are affected by heat. An increase in growth temperature from 22 to 30°C effectively inhibited S-PTGS and A-PTGS but not IR-PTGS in *Arabidopsis* (Zhong et al., 2013). The warmth-induced PTGS release most likely occured during a critical step that leads to the formation of stable dsRNAs involving RDR6 and SUPPRESSOR OF GENE SILENCING 3 (SGS3). The abundance of many endogenous tasiRNAs was also significantly reduced by the 30°C growth, consistent with increased transcript levels of *TAS* and tasiRNA-target genes, which may affect the morphological acclimation (**Figure 1A**). The temperature increase reduced the protein abundance of SGS3, as a consequence, attenuating the formation of stable dsRNAs. Overexpression of SGS3 could release such warmth-triggered inhibition of siRNA biogenesis (Zhong et al., 2013). Heat shock (37°C for 1 h) also decreased the accumulation of *TAS1*-derived siRNAs, whereas their target genes *HEAT-INDUCED TAS1 TARGET1* (*HTT1*) and *HTT2* were highly up-regulated by heat shock (Li et al., 2014b). Meanwhile, *HTT1* and *HTT2* were directly activated by HsfA1a through binding to their promoters. HTT1 mediated thermotolerance by acting as a cofactor of Hsp70-14 complexes (Li et al., 2014b; **Figure 1D**). Some nat-siRNAs are responsive to heat stress in *Brassica rapa* and *Arabidopsis*. For example, nat-siRNAs derived from Bra018216/Bra018217 and its homologous NAT pair AT3G46230/AT3G46220 were induced by heat, leading to the induction of the former gene (*Bra018216* and *AT3G46230*) and the repression of the latter gene (*Bra018217* and *AT3G46220*; Yu et al., 2013; **Figure 1D**). A novel class of heat-responsive small RNAs derived from the chloroplast genome of *Brassica rapa* has been reported (Wang et al., 2011). Many members of chloroplast small RNAs (csRNAs) families are highly sensitive to heat stress, and some csRNAs respond to heat stress by silencing target genes (Wang et al., 2011). It will be interesting to investigate the role of these csRNAs in RPS1-mediated heat-responsive retrograde pathway (**Figure 1D**). Although ra-siRNAs-mediated RdDM pathway is required for basal thermotolerance (Popova et al., 2013), the underlying mechanism is still not clear (**Figure 1D**). The study of *ONSEN* reveals a novel regulation mechanism via siRNAs in heat responses (Ito et al., 2011). In *Arabidopsis* seedlings, *ONSEN* is transiently activated by heat stress and re-silenced during the recovery period. A surprisingly high frequency of retrotransposition, which produces new *ONSEN* insertions, is observed in the progeny of stressed *nrpd1* plants but not of the wild-type plants, suggesting a crucial role of the ra-siRNA pathway in restricting transgenerational retrotransposition triggered by heat stress. Moreover, natural and experimentally induced variants in endogenous loci harboring new *ONSEN* insertions confer heat responsiveness to nearby genes. Therefore, heat-induced mobility bursts of *ONSEN* may generate novel, stress-responsive regulatory gene networks (Ito et al., 2011). A recent study in maize has also demonstrated that allelic variation for insertions of the TEs associated with heat stress-responsive expression can contribute to variation in the regulation of nearby genes, probably by providing binding sites for transcription factors or influencing chromatin (Makarevitch et al., 2015). The roles of endogenous IR-derived siRNAs and diRNAs in heat responses remain to be investigated.

High temperatures often enhance the VIGS-mediated disease resistance (**Figure 1C**). The temperature shift from 25 to 30°C induced the accumulation of siRNAs and increases the cassava geminivirus-induced RNA silencing in plants (Chellappan et al., 2005). Temperature-dependent survival of *Turnip crinkle virus*-infected *Arabidopsis* plants relies on an RNA silencing-based defense that requires DCL2, AGO2, and HEN1 (Zhang et al., 2012). The activity of DCL2 was up-regulated by high temperature, suggesting that DCL2 protein may be a temperature-sensitive component responsible for modulation of RNA silencing pathway (Zhang et al., 2012). In addition, RDR6 may be closely related to the temperature sensitivity of the silencing pathway in *Nicotiana benthamiana* (Qu et al., 2005). Plants with reduced expression of NbRDR6 were more susceptible to various viruses and this effect was more pronounced at higher growth temperatures (Qu et al., 2005). Moreover, NbRDR6 plays a root-specific role in the inhibition of *Chinese wheat mosaic virus* (CWMV) accumulation and biogenesis of CWMV siRNAs at higher temperatures (Andika et al., 2013). It will be interesting to investigate whether other components of RNA silencing also affect virus resistance under high temperatures.

### Other Epigenetic Regulation of Heat Responses

In recent years, new epigenetic mechanisms have been revealed to regulate the heat responses, including lncRNAs, HEAT INTOLERANT 4 (HIT4)-mediated non-canonical TGS regulation and other two unorthodox pathways. LncRNAs, with size larger than 200 nt, are precursors for siRNA biogenesis and act as scaffolds for the establishment of DNA methylation and histone modifications (Wierzbicki, 2012). In *Arabidopsis*, the expression of *HSFB2a* was counteracted by a natural and heat-inducible long non-coding antisense RNA, *asHSFB2a* (Wunderlich et al., 2014). In leaves, the antisense RNA gene was only expressed after heat stress and dependent on the activity of HSFA1a/HSFA1b. *HSFB2a* and *asHSFB2a* RNAs were also present in the absence of heat stress in the female gametophyte. HSFB2a activity temporarily repressed vegetative growth during development and after heat stress, the antisense regulation by *asHSFB2a* counteracted this effect to restore growth and further development (**Figure 1D**; Wunderlich et al., 2014). Other 15 heat-responsive lncRNAs have been found in *Arabidopsis*, but their functions are still unknown (Di et al., 2014). Seventy-seven putative heat-responsive lncRNAs, which are not conserved among plant species, have been identified in wheat (Xin et al., 2011). Among them, TahlnRNA27 and TalnRNA5 are the precursors of *tae-miR2010* and *tae-miR2004*, respectively. These two lnRNAs and miRNAs were up-regulated after heat stress in heat sensitive genotype Chinese Spring (CS) and heat tolerant genotype TAM107. Nine heat-responsive lncRNAs such as TalnRNA21 may be precursors of siRNAs. TalnRNA9 and TalnRNA12 are identified as signal recognition particle (SRP) 7S RNA variants and can be regulated by siRNAs. Besides, three lncRNAs (TahlnRNA12, TahlnRNA23, and TahlnRNA29) are characterized as U3 snoRNAs (Xin et al., 2011). Interestingly, lnc-508 was down-regulated by heat and cold, while lnc-168 was down-regulated by heat and salt in *Arabidopsis* (Di et al., 2014). Twenty-three lncRNAs respond to both powdery mildew infection and heat stress in wheat (Xin et al., 2011). These results suggest that like miRNAs, lncRNAs may also integrate the regulatory networks of heat stress with that of other biotic and abiotic stresses.

HIT4 has been reported to mediate heat-induced decondensation of chromocenters and release from TGS with no change in the level of DNA methylation (Wang et al., 2013, 2014). HIT4 acts independent of MOM1 at the level of heterochromatin organization and this activity is essential for basal thermotolerance in plants. Thus, HIT4 delineates a novel TGS regulation pathway, involving a currently unidentified component that links HIT4 relocation and the large-scale reorganization of chromatin (Wang et al., 2013, 2014). A special inductive temperature shift released the heterochromatin-associated silencing in *Arabidopsis* plants in a genome-wide manner (Tittel-Elmer et al., 2010). This occurred without alteration of repressive epigenetic modifications and did not involve common epigenetic mechanisms. Such induced release of silencing was rapidly restored, without the involvement of factors known to be required for silencing initiation. Therefore, stress-induced destabilization of heterochromatic TGS and its re-establishment may involve novel mechanisms that repress transcription (Tittel-Elmer et al., 2010). In a recent study, long-term heat stress activated the *Arabidopsis* imprinted gene *SUPPRESSOR OF DRM1 DRM2 CMT3* (*SDC*), which encodes a putative F-Box protein and is silent during vegetative growth due to DNA methylation (Sanchez and Paszkowski, 2014). The heat-mediated transcriptional induction of *SDC* occurred only above a particular window of absolute temperature and was proportional to the level of stress. After heat stress, *SDC* was slowly re-silenced, allowing a temporal extension of SDC activity to contribute to the recovery of plant biomass. The *SDC* activation seems to occur independently and in parallel to canonical heat-shock perception and signaling, but rely on a yet undefined epigenetic mechanism (Sanchez and Paszkowski, 2014).

## Transgenerational Memory and Evolutionary Adaptation

Transgenerational memory, also known as epigenetic inheritance, refers to the transmittance of epigenetic states and certain environmental responses from one generation to the next. These transgenerational effects may offer the offspring an adaptive advantage or genomic flexibility for better fitness. Recent evidence suggests that some abiotic and biotic stress responses are transgenerational in plants. For example, exposure of *Arabidopsis* plants to UV-C and flagellin can induce transgenerational increases in HRF (Molinier et al., 2006). Heat responses also exhibit transgenerational epigenetic inheritance (Boyko et al., 2010; Lang-Mladek et al., 2010; Ito et al., 2011; Zhong et al., 2013; Iwasaki and Paszkowski, 2014; Migicovsky et al., 2014). The immediate progeny of heat-stressed *Arabidopsis* plants have fewer, but larger leaves, and tend to bolt earlier (Migicovsky et al., 2014). These plants have increased expression of *HSFA2*, but reduced expression of *ROS1* and several Su(var)3–9 homologs (*SUVH*) genes involved in H3K9 methylation and DNA methylation. These phenotypic and epigenetic changes are partially deficient in the offspring of heat-stressed *dcl2* and *dcl3* mutants (Migicovsky et al., 2014). It is also reported that transgenerational adaptation of *Arabidopsis* to stress requires DNA methylation and the function of Dicer-like proteins (Boyko et al., 2010). However, the transgenerational retrotransposition of *ONSEN* is prevented by the siRNAs pathway (Matsunaga et al., 2012), while SGS3 overexpression could decrease the warmth-induced transgenerational memory (Zhong et al., 2013). Thus, the role of siRNAs pathway in transgenerational memory of heat responses remains controversial. The involvement of AGO1 and the *miR156-SPL* pathway has been demonstrated to maintain the short memory of acquired thermotolerance in the adaptation to recurring heat stress at the physiological and molecular level in *Arabidopsis* (Stief et al., 2014). *Bra-miR168* and its target *braAGO1* are also suggested to be putative messengers that mediate meiotic epigenetic inheritance in *Brassica rapa* (Bilichak et al., 2015). Further experiments on transgenerational heat stress in the hypomorphic *ago1* mutants may shade a new light on the contribution of AGO1 and the miRNA pathway to epigenetic inheritance in plants.

The role of DNA methylation in transgenerational memory is also obscure. It is reported that there seems no consistent correlation between DNA methylation changes of transgene and the warmth-induced transgenerational release of PTGS (Zhong et al., 2013). Similarly, DNA methylation may be also not involved in the release of transgene TGS by heat stress (Lang-Mladek et al., 2010; Pecinka et al., 2010; Tittel-Elmer et al., 2010). However, it remains a possibility that changes in DNA methylation at certain sites of a silenced target gene or at certain loci of the genome are responsible for the transgenerational memory, as CG methylation plays a central role in transgenerational stability of the *Arabidopsis* epigenome (Mathieu et al., 2007). Loss of CG methylation triggers genome-wide aberrant *de novo* non-CG methylation by interfering with the RdDM process and expression of DNA demethylases, as well as progressive H3K9 remethylation of heterochromatin. It is proposed that immediate, non-heritable stress responses may be associated with alteration of non-CG methylation patterns mediated by siRNAs/RdDM and ROS1/DME, while long-term, heritable adaptation to a changing environment would require modulation of CG patterns (Mathieu et al., 2007). Recently, a forward genetic screen revealed that DDM1 and MOM1 act redundantly in preventing the transmission of stress-induced transcriptional changes to progeny of the stressed plants (Iwasaki and Paszkowski, 2014). Such DDM1- and MOM1-mediated or other mechanisms of chromatin resetting could prevent the transgenerational transmission of environmentally induced epigenetic traits. The roles of other epigenetic mechanisms in transgenerational memory, such as H2A.Z and H3K27me3, are worth further investigation.

Although the mechanisms remain to be elucidated, the transgenerational memory of heat responses may contribute to evolutionary adaptation. The warmth-induced epigenetic memory was maintained for at least three generations with gradually declining (Zhong et al., 2013). Heat stress also induced transgenerational phenotypic changes over three generations (Suter and Widmer, 2013a,b). Ancestral exposure to elevated temperatures over P and F1 generations resulted in increased fitness in the F3 heat-treated *Arabidopsis* plants (Whittle et al., 2009). Thus, the transgenerational memory of heat responses may allow potentially long-term adaptation and rapid evolution, as chromatin modifications can be mitotically or meiotically heritable. Stress-induced epigenetic changes may lead to the formation of heritable epialleles and transcriptional activation of TEs (Mirouze and Paszkowski, 2011). The epialleles and transposon-driven variation in gene expression may contribute to the phenotypic diversity of different individuals in a population or a species that can be subject to natural selection (Mirouze and Paszkowski, 2011). The transgenerational retrotransposition of *ONSEN* may reshape gene regulatory networks and potentially create novel traits for adaptation to heat stress, as genes in the vicinity of *ONSEN* neo-insertions aquired heat-responsiveness (Ito et al., 2011). Given that the activity of transposable element is important for adaptive plant evolution (Lisch, 2013), and *ONSEN* is evolutionary conserved and transcriptionally activated by environmental heat stress in some Brassicaceae species (Ito et al., 2013), it will be interesting to explore the possible role of *ONSEN* and other TEs in plant evolution. Whether heat mediates the formation of heritable epialleles still needs to be investigated. Random RdDM-mediated epiallele formation is suggested to play a greater role in evolution than genetic variation (Matzke and Mosher, 2014). Epigenetic variation in DNA methylation among epigenetic recombinant inbred lines (epiRILs) that are nearly isogenic but highly variable at the level of DNA methylation, can cause substantial heritable variation of drought tolerance and nutrient plasticity (Zhang et al., 2013). It will be worth investigating whether heat-induced changes of global methylation creates potential for the evolution of phenotypic plasticity.

## Concluding Remarks

Heat greatly affects the growth, development, and productivity of plants. Several heat sensors have been reported, including the calcium channel in the plasma membrane, H2A.Z-containing nucleosomes in the nucleus, and two unfolded protein sensors in ER and the cytosol. Importantly, different epigenetic regulations may also be involved in the responses to different heat treatments (**Table 1**). The epigenetic regulation of warm and high temperatures mainly involves warmth-induced PTGS release, enhanced VIGS-mediated resistance, and H2A.Z-mediated morphological acclimation and acceleration of flowering. Various epigenetic mechanisms (known or unknown) are involved in response to heat stress. It is notable that different lengths (from 1 h to 4 days) of heat treatment at 37°C have diverse effects on the epigenome, suggesting the complexity in the epigenetic regulation of heat stress.

Despite recent advances in our understanding of the genetic and epigenetic mechanisms involved in heat stress sensing in plants, many questions remain to be answered by future research (**Box 1**). Perhaps the most important questions in the genetic mechanisms of heat responses are: what are the primary heat sensors? In addition to the four heat sensors mentioned above, a list of other components like mRNAs, miRNAs and hormonal import and antiport channels may also be plausible thermometers based on physical capacities (McClung and Davis, 2010). Systematic analysis of the changes in the genome, transcriptome, microme, and proteome by omic approaches may help to identify novel transcriptional, translational, and posttranslational regulation components and underlying mechanisms in plant heat responses (Bokszczanin and Fragkostefanakis, 2013; Hasanuzzaman et al., 2013). Perhaps plants sense heat through different organelles in different phases of the response, and then these signaling pathways are integrated and work synergisticly or differentially to defend plants from heat-induced deleterious effects.

**Box 1. Proposals of future researches.**

• What are the primary heat sensors? Are the CNGCs, H2A.Z or the unfolded protein sensors the true heat sensor?

• How are the different heat-sensing pathways integrated in plant cells?

• What are the roles of the RdDM pathway in response to heat in crop plants?

• How do the different histone modifications regulate the heat responses in different plants?

• Do histone modifications regulate the response of circadian clock to high temperature and heat stress?

• By which precise mechanism is the H2A.Z occupancy regulated by high temperature? Is H2A.Z occupancy regulated by heat stress? Is H2A.Z occupancy involved in the modulation of plant immunity and circadian clock by high temperature?

• How are the heat-responsive miRNAs, siRNAs and lncRNAs regulated by heat? What are the regulatory networks of their targets in plants?

• How is the epigenetic regulation of heat responses integrated with the epigenetic regulation of other biotic and abiotic responses?

• How is the transgenerational memory of heat responses controlled?

• How to improve the thermotolerance of crops without sacrificing growth?

As listed in **Box 1**, a set of questions concerning the epigenetic regulations of heat responses are proposed. One of the major challenge ahead may be to discover the mechanisms of transgenerational memory heat responses. Systemic screening for factors regulating transgenerational memory of heat may address the long-term controversial issue. Besides, the roles of DNA methylation and histone modifications in response to heat need to be defined. miRNAs, lncRNAs and the chromatin-remodeling gene *AtCHR12* have been suggested to integrate the epigenetic regulation of heat stress with the regulation of other biotic and abiotic stresses. Other epigenetic regulations may also have similar functions. Further investigations should be focused on the epigenetic regulatory networks between heat stress and other biotic and abiotic stresses. We should note that most experiments on the role of genetic and epigenetic regulation in heat responses are limited to the model *Arabidopsis* plants in laboratory conditions with short-term heat treatment. As temperatures in the field change seasonally and fluctuate daily, further studies should also be centered on the genetic and epigenetic regulations of heat responses in crop plants in the field, which may produce practical approaches to develop crop plants with improved thermotolerance.

## Acknowledgment

This work was supported by grants from the National Research Program of China (2011CB100700).
